# BINGE EATING AMONG OLDER WOMEN: PREVALENCE RATES AND HEALTH CORRELATES ACROSS THREE UNIQUE SAMPLES

**DOI:** 10.1093/geroni/igz038.3375

**Published:** 2019-11-08

**Authors:** Lisa S Kilpela, Carolyn B Becker, Francesca Gomez, Keesha Middlemass, Sara E Espinoza, Nicolas Musi

**Affiliations:** 1 UT Health San Antonio, San Antonio, Texas, United States; 2 Trinity University, San Antonio, Texas, United States; 3 Howard University, Washington, District of Columbia, United States; 4 Barshop Institute for Longevity & Aging Studies, University of Texas Health Science Center, San Antonio, Texas, United States

## Abstract

Emerging research indicates that older women struggle with disordered eating more frequently than once thought. Among older women, binge eating (BE; consuming unusually large amounts of food in one siting while feeling a loss of control) appears to be the most common form of disordered eating. Notably, BE is associated with significant medical morbidity, including metabolic dysfunction and chronic pain. Aging-related experiences, such as sleep disruptions following menopause, mood changes and psychosocial stressors, may increase risk for BE. This study comprises a 3-sample comparison of BE prevalence and health correlates among older women. We gathered self-reported frequencies of BE in three separate samples of older women, using three different methods and validated measures. Sample 1: N = 185 women aged 60-83; collected online via snowball sampling and Amazon MTurk; 86% White. Sample 2: N = 100 women aged 55-79; collected online via snowball sampling; 72% White; 50% Masters/Doctoral Degree; 72% married. Sample 3: N = 64 women aged 66+ living with food insecurity, collected in person at local food pantries; 65% Hispanic, 16% African American; 39% disabled status; 48% < high school/GED; 47% household income < $10,000/year. Per DSM-5 frequency criterion of BE at least weekly, we found prevalence rates ranging from 19%-26.5% across the samples; correlates included elevated negative mood, worry, and BMI, and less nutritious food consumption. Across three very different samples in terms of race/ethnicity, education, and food security status, we found consistent rates of self-reported BE at least weekly (roughly 1/5). Implications will be discussed.

